# Ethyl 1-*sec*-butyl-2-phenyl-1*H*-benzimidazole-5-carboxyl­ate

**DOI:** 10.1107/S160053681000872X

**Published:** 2010-03-13

**Authors:** Natarajan Arumugam, Aisyah Saad Abdul Rahim, Shafida Abd Hamid, Madhukar Hemamalini, Hoong-Kun Fun

**Affiliations:** a School of Pharmaceutical Sciences, Universiti Sains Malaysia, 11800 USM, Penang, Malaysia; b Kulliyyah of Science, International Islamic University Malaysia (IIUM), Jalan Istana, Bandar Indera Mahkota, 25200 Kuantan, Pahang, Malaysia; c X-ray Crystallography Unit, School of Physics, Universiti Sains Malaysia, 11800 USM, Penang, Malaysia

## Abstract

In the title mol­ecule, C_20_H_22_N_2_O_2_, the benzimidazole ring system is essentially planar, with a maximum deviation of 0.024 (1) Å. The dihedral angle between the phenyl and benzimidazole ring system is 43.71 (5)°. The atoms of the butyl group are disordered over two sets of sites with occupancies of 0.900 (4) and 0.100 (4). In the crystal structure, mol­ecules are connected by weak inter­molecular C—H⋯O hydrogen bonds, forming chains along the *b* axis. The crystal structure is further stabilized by C—H⋯π inter­actions.

## Related literature

For background to the applications of benzimidazole compounds, see: Spasov *et al.* (1999 ▶); Grassmann *et al.* (2002 ▶); Evans *et al.* (1997 ▶); White *et al.* (2004 ▶); Demirayak *et al.* (2002 ▶). For details of hydrogen bonding, see: Jeffrey & Saenger (1991 ▶); Jeffrey (1997 ▶); Scheiner (1997 ▶). For the stability of the temperature controller used in the data collection, see: Cosier & Glazer (1986 ▶).
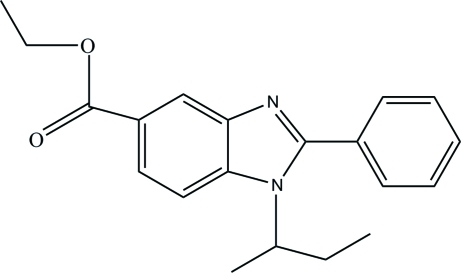


## Experimental

### 

#### Crystal data

C_20_H_22_N_2_O_2_
*M* *_r_* = 322.40
Monoclinic, 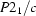

*a* = 9.9926 (7) Å
*b* = 12.3287 (11) Å
*c* = 13.9635 (12) Å
β = 93.120 (3)°
*V* = 1717.7 (2) Å^3^
*Z* = 4
Mo *K*α radiation
μ = 0.08 mm^−1^
*T* = 100 K
0.36 × 0.17 × 0.16 mm

#### Data collection

Bruker APEX DUO CCD area-detector diffractometer
Absorption correction: multi-scan (*SADABS*; Bruker, 2009 ▶) *T*_min_ = 0.972, *T*_max_ = 0.987
22823 measured reflections
6168 independent reflections
4186 reflections with *I* > 2σ(*I*)
*R*_int_ = 0.055

#### Refinement

*R*[*F* ^2^ > 2σ(*F* ^2^)] = 0.053
*wR*(*F* ^2^) = 0.168
*S* = 1.06
6168 reflections
239 parameters
H-atom parameters constrained
Δρ_max_ = 0.46 e Å^−3^
Δρ_min_ = −0.30 e Å^−3^

### 

Data collection: *APEX2* (Bruker, 2009 ▶); cell refinement: *SAINT* (Bruker, 2009 ▶); data reduction: *SAINT*; program(s) used to solve structure: *SHELXTL* (Sheldrick, 2008 ▶); program(s) used to refine structure: *SHELXTL*; molecular graphics: *SHELXTL*; software used to prepare material for publication: *SHELXTL* and *PLATON* (Spek, 2009 ▶).

## Supplementary Material

Crystal structure: contains datablocks global, I. DOI: 10.1107/S160053681000872X/lh5008sup1.cif

Structure factors: contains datablocks I. DOI: 10.1107/S160053681000872X/lh5008Isup2.hkl

Additional supplementary materials:  crystallographic information; 3D view; checkCIF report

## Figures and Tables

**Table 1:** Hydrogen-bond geometry (Å, °) *Cg*1 is the centroid of the N1/N2/C7–C8/C13 ring.

*D*—H⋯*A*	*D*—H	H⋯*A*	*D*⋯*A*	*D*—H⋯*A*
C9—H9*A*⋯O1^i^	0.93	2.45	3.3781 (17)	172
C20*A*—H20*B*⋯O1^i^	0.96	2.56	3.419 (2)	148
C19*A*—H19*B*⋯*Cg*1	0.96	2.80	3.3793 (17)	120

Symmetry code: (i) 
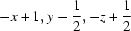
.

**Fig. 1.**
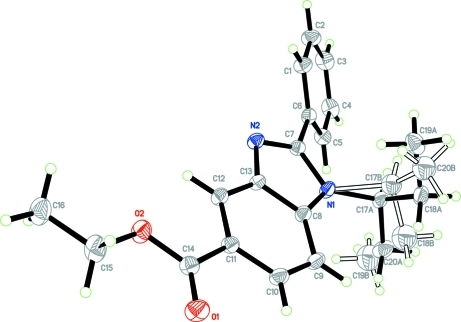
The asymmetric unit of the title compound, showing 50% probability displacement ellipsoids and the atom-numbering scheme. The minor disorder component is shown with open bonds.

**Fig. 2.**
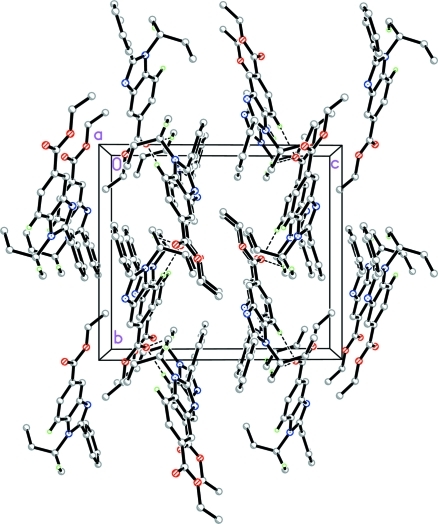
The crystal packing of the title compound, showing hydrogen bonds as dashed lines. H atoms are not involved in the hydrogen bond interactions are omitted for clarity.

## supplementary crystallographic information

### Comment

The benzimidazole motif is an important pharmacophore in drug discovery (Spasov *et al.*, 1999). Substituted benzimidazole derivatives have diverse therapeutic applications as they exhibit antihistamine (Grassmann *et al.*, 2002), anti-HIV-1 (Evans *et al.*,  1997), antitumour (White *et al.*, 2004) and potential anticancer activities (Demirayak *et al.*, 2002). In view of their importance in the field of drug discovery, the crystal structure determination of the title compound was carried out and the results are presented here.

In the asymmetric unit of the title compound (Fig. 1), the benzimidazole ring system is essentially planar with a maximum deviation of 0.024 (1)Å for atom N2. The butyl group is disordered over two sites with occupancies of 0.900 (4) and 0.100 (4). The dihedral angle between the benzimidazole ring system (N1–N2/C7–C13) and the phenyl ring (C1–C6) is 43.71 (5)° . In the crystal structure (Fig. 2),  molecules are connected by weak intermolecular C9—H9A···O1^i^ and C20A—H20B···O1^i^ (see Table 1 for symmetry codes) hydrogen bonds, forming one-dimensional chains along the b-axis. The crystal structure is further stabilized by C—H···π interactions (Table 1), involving N1–N2/C7–C8/C13 (centroid Cg1).

### Experimental

A solution of ethyl-3-amino-4-(*sec*-butylamino) benzoate (200 mg, 0.84 mmol) and sodium bisulfite adduct of benzaldehyde (353 mg, 1.68 mmol)  in DMF was treated under microwave conditions at 130°C  for 2 minutes. The reaction mixture was then diluted in EtOAc (20 mL) and washed with H_2_O (20 mL). The organic layer was collected and dried over Na_2_SO_4_. The solvent was removed under reduced pressure to afford the crude product, which upon recrystallisation from EtOAc, revealed the title compound as colourless crystals.

### Refinement

All hydrogen atoms were positioned geometrically [C–H = 0.93 or 0.97Å] and were refined using a riding model, with *U*_iso_(H) = 1.2 or 1.5 *U*_eq_(C). A rotating group model was applied to the methyl groups. The butyl group is disordered over two sites with refined occupancies of 0.900 (4) and 0.100 (4).

### Crystal data

**Table:**

C_20_H_22_N_2_O_2_	*F*(000) = 688
*M**_r_* = 322.40	*D*_x_ = 1.247 Mg m^−^^3^
Monoclinic, *P*2_1_/*c*	Mo *K*α radiation, λ = 0.71073 Å
Hall symbol: -P 2ybc	Cell parameters from 3770 reflections
*a* = 9.9926 (7) Å	θ = 2.9–32.2°
*b* = 12.3287 (11) Å	µ = 0.08 mm^−^^1^
*c* = 13.9635 (12) Å	*T* = 100 K
β = 93.120 (3)°	Block, colourless
*V* = 1717.7 (2) Å^3^	0.36 × 0.17 × 0.16 mm
*Z* = 4	

### Data collection

**Table:**

Bruker APEX DUO CCD area-detector diffractometer	6168 independent reflections
Radiation source: fine-focus sealed tube	4186 reflections with *I* > 2σ(*I*)
graphite	*R*_int_ = 0.055
φ and ω scans	θ_max_ = 32.5°, θ_min_ = 2.6°
Absorption correction: multi-scan (*SADABS*; Bruker, 2009)	*h* = −15→14
*T*_min_ = 0.972, *T*_max_ = 0.987	*k* = −17→18
22823 measured reflections	*l* = −21→18

### Refinement

**Table:**

Refinement on *F*^2^	Primary atom site location: structure-invariant direct methods
Least-squares matrix: full	Secondary atom site location: difference Fourier map
*R*[*F*^2^ > 2σ(*F*^2^)] = 0.053	Hydrogen site location: inferred from neighbouring sites
*wR*(*F*^2^) = 0.168	H-atom parameters constrained
*S* = 1.06	*w* = 1/[σ^2^(*F*_o_^2^) + (0.0891*P*)^2^ + 0.1439*P*] where *P* = (*F*_o_^2^ + 2*F*_c_^2^)/3
6168 reflections	(Δ/σ)_max_ = 0.001
239 parameters	Δρ_max_ = 0.46 e Å^−^^3^
0 restraints	Δρ_min_ = −0.30 e Å^−^^3^

### Special details

**Table:**

Experimental. The crystal was placed in the cold stream of an Oxford Cryosystems Cobra open-flow nitrogen cryostat (Cosier & Glazer, 1986) operating at 100.0 (1) K.
Geometry. All esds (except the esd in the dihedral angle between two l.s. planes) are estimated using the full covariance matrix. The cell esds are taken into account individually in the estimation of esds in distances, angles and torsion angles; correlations between esds in cell parameters are only used when they are defined by crystal symmetry. An approximate (isotropic) treatment of cell esds is used for estimating esds involving l.s. planes.
Refinement. Refinement of F^2^ against ALL reflections. The weighted R-factor wR and goodness of fit S are based on F^2^, conventional R-factors R are based on F, with F set to zero for negative F^2^. The threshold expression of F^2^ > 2sigma(F^2^) is used only for calculating R-factors(gt) etc. and is not relevant to the choice of reflections for refinement. R-factors based on F^2^ are statistically about twice as large as those based on F, and R- factors based on ALL data will be even larger.

### Fractional atomic coordinates and isotropic or equivalent isotropic displacement parameters (Å^2^)

**Table:**

	*x*	*y*	*z*	*U*_iso_*/*U*_eq_	Occ. (<1)
O1	0.48537 (10)	0.96613 (8)	0.17446 (8)	0.0271 (2)	
O2	0.65687 (10)	1.02815 (8)	0.09245 (8)	0.0254 (2)	
N1	0.90319 (11)	0.57520 (9)	0.18622 (8)	0.0193 (2)	
N2	0.99597 (11)	0.71445 (9)	0.10769 (8)	0.0182 (2)	
C1	1.25733 (14)	0.59498 (11)	0.13359 (10)	0.0227 (3)	
H1A	1.2658	0.6666	0.1538	0.027*	
C2	1.37107 (15)	0.53521 (12)	0.11435 (11)	0.0268 (3)	
H2A	1.4553	0.5672	0.1213	0.032*	
C3	1.35904 (15)	0.42848 (12)	0.08494 (11)	0.0276 (3)	
H3A	1.4353	0.3887	0.0726	0.033*	
C4	1.23430 (16)	0.38062 (12)	0.07376 (10)	0.0259 (3)	
H4A	1.2267	0.3087	0.0541	0.031*	
C5	1.12036 (15)	0.43963 (11)	0.09179 (10)	0.0216 (3)	
H5A	1.0364	0.4074	0.0834	0.026*	
C6	1.13109 (13)	0.54764 (10)	0.12256 (9)	0.0182 (2)	
C7	1.01178 (13)	0.61389 (10)	0.13933 (9)	0.0172 (2)	
C8	0.81056 (13)	0.65853 (10)	0.18441 (9)	0.0184 (2)	
C9	0.68265 (14)	0.66727 (11)	0.21995 (10)	0.0222 (3)	
H9A	0.6440	0.6104	0.2524	0.027*	
C10	0.61687 (14)	0.76473 (11)	0.20428 (10)	0.0213 (3)	
H10A	0.5318	0.7735	0.2271	0.026*	
C11	0.67404 (13)	0.85123 (10)	0.15492 (9)	0.0179 (2)	
C12	0.80110 (13)	0.84152 (10)	0.11974 (9)	0.0179 (2)	
H12A	0.8389	0.8982	0.0865	0.021*	
C13	0.87006 (13)	0.74459 (10)	0.13564 (9)	0.0165 (2)	
C14	0.59468 (14)	0.95227 (10)	0.14299 (9)	0.0200 (3)	
C15	0.58654 (17)	1.13040 (11)	0.08026 (12)	0.0296 (3)	
H15A	0.5857	1.1685	0.1410	0.036*	
H15B	0.4946	1.1180	0.0569	0.036*	
C16	0.65916 (17)	1.19568 (12)	0.00926 (12)	0.0325 (3)	
H16A	0.6164	1.2650	0.0006	0.049*	
H16B	0.6572	1.1580	−0.0510	0.049*	
H16C	0.7505	1.2059	0.0325	0.049*	
C17A	0.90159 (17)	0.47759 (12)	0.24809 (13)	0.0195 (3)	0.900 (4)
H17A	0.9893	0.4426	0.2449	0.023*	0.900 (4)
C18A	0.88752 (18)	0.51121 (14)	0.35205 (12)	0.0246 (4)	0.900 (4)
H18A	0.7971	0.5372	0.3595	0.030*	0.900 (4)
H18B	0.9016	0.4483	0.3931	0.030*	0.900 (4)
C19A	0.98655 (19)	0.59931 (16)	0.38395 (11)	0.0279 (4)	0.900 (4)
H19A	0.9823	0.6110	0.4517	0.042*	0.900 (4)
H19B	0.9644	0.6654	0.3503	0.042*	0.900 (4)
H19C	1.0755	0.5772	0.3700	0.042*	0.900 (4)
C20A	0.79610 (19)	0.39498 (14)	0.21182 (18)	0.0290 (4)	0.900 (4)
H20A	0.8087	0.3786	0.1457	0.044*	0.900 (4)
H20B	0.7082	0.4247	0.2179	0.044*	0.900 (4)
H20C	0.8053	0.3298	0.2492	0.044*	0.900 (4)
C17B	0.931 (2)	0.4995 (15)	0.2836 (15)	0.030 (4)*	0.100 (4)
H17B	1.0131	0.4586	0.2748	0.035*	0.100 (4)
C18B	0.815 (2)	0.4191 (17)	0.2711 (17)	0.046 (5)*	0.100 (4)
H18C	0.8371	0.3558	0.3101	0.055*	0.100 (4)
H18D	0.7362	0.4521	0.2965	0.055*	0.100 (4)
C19B	0.780 (3)	0.383 (2)	0.1742 (19)	0.051 (7)*	0.100 (4)
H19D	0.7015	0.3376	0.1743	0.077*	0.100 (4)
H19E	0.8529	0.3420	0.1505	0.077*	0.100 (4)
H19F	0.7618	0.4445	0.1336	0.077*	0.100 (4)
C20B	0.944 (2)	0.5464 (19)	0.3761 (14)	0.037 (4)*	0.100 (4)
H20D	1.0175	0.5132	0.4120	0.055*	0.100 (4)
H20E	0.8628	0.5351	0.4086	0.055*	0.100 (4)
H20F	0.9601	0.6228	0.3704	0.055*	0.100 (4)

### Atomic displacement parameters (Å^2^)

**Table:**

	*U*^11^	*U*^22^	*U*^33^	*U*^12^	*U*^13^	*U*^23^
O1	0.0215 (5)	0.0248 (5)	0.0358 (6)	0.0061 (4)	0.0090 (4)	0.0004 (4)
O2	0.0251 (5)	0.0176 (4)	0.0341 (5)	0.0065 (4)	0.0076 (4)	0.0053 (4)
N1	0.0176 (5)	0.0167 (5)	0.0242 (5)	0.0018 (4)	0.0060 (4)	0.0052 (4)
N2	0.0164 (5)	0.0170 (5)	0.0217 (5)	0.0007 (4)	0.0046 (4)	0.0004 (4)
C1	0.0199 (7)	0.0207 (6)	0.0276 (6)	0.0008 (5)	0.0039 (5)	−0.0009 (5)
C2	0.0182 (7)	0.0296 (7)	0.0327 (7)	0.0025 (5)	0.0037 (5)	0.0001 (6)
C3	0.0243 (7)	0.0305 (7)	0.0286 (7)	0.0105 (6)	0.0047 (5)	−0.0016 (5)
C4	0.0304 (8)	0.0229 (6)	0.0244 (6)	0.0086 (6)	0.0013 (5)	−0.0035 (5)
C5	0.0222 (7)	0.0200 (6)	0.0224 (6)	0.0020 (5)	0.0008 (5)	−0.0017 (4)
C6	0.0184 (6)	0.0186 (5)	0.0180 (5)	0.0030 (5)	0.0037 (4)	0.0014 (4)
C7	0.0157 (6)	0.0178 (5)	0.0182 (5)	0.0013 (4)	0.0026 (4)	0.0005 (4)
C8	0.0171 (6)	0.0165 (5)	0.0217 (6)	0.0007 (4)	0.0032 (5)	0.0026 (4)
C9	0.0172 (6)	0.0209 (6)	0.0289 (7)	−0.0007 (5)	0.0056 (5)	0.0058 (5)
C10	0.0157 (6)	0.0213 (6)	0.0272 (6)	0.0008 (5)	0.0054 (5)	0.0017 (5)
C11	0.0165 (6)	0.0164 (5)	0.0209 (6)	0.0009 (4)	0.0018 (4)	0.0001 (4)
C12	0.0181 (6)	0.0154 (5)	0.0205 (6)	−0.0002 (4)	0.0040 (5)	0.0002 (4)
C13	0.0159 (6)	0.0150 (5)	0.0190 (5)	−0.0009 (4)	0.0036 (4)	0.0002 (4)
C14	0.0192 (6)	0.0186 (5)	0.0223 (6)	0.0016 (5)	0.0020 (5)	−0.0010 (4)
C15	0.0331 (8)	0.0192 (6)	0.0373 (8)	0.0110 (6)	0.0090 (6)	0.0046 (5)
C16	0.0369 (9)	0.0224 (6)	0.0387 (8)	0.0056 (6)	0.0075 (7)	0.0064 (6)
C17A	0.0188 (7)	0.0150 (6)	0.0249 (8)	0.0014 (5)	0.0035 (6)	0.0065 (6)
C18A	0.0219 (8)	0.0305 (8)	0.0220 (7)	0.0040 (7)	0.0056 (6)	0.0087 (6)
C19A	0.0277 (9)	0.0365 (10)	0.0194 (7)	0.0063 (8)	0.0005 (6)	−0.0018 (6)
C20A	0.0262 (9)	0.0185 (7)	0.0426 (13)	−0.0041 (6)	0.0040 (8)	0.0028 (8)

### Geometric parameters (Å, °)

**Table:**

O1—C14	1.2114 (16)	C15—C16	1.495 (2)
O2—C14	1.3443 (16)	C15—H15A	0.9700
O2—C15	1.4488 (16)	C15—H15B	0.9700
N1—C8	1.3821 (16)	C16—H16A	0.9600
N1—C7	1.3824 (16)	C16—H16B	0.9600
N1—C17A	1.4820 (17)	C16—H16C	0.9600
N1—C17B	1.660 (19)	C17A—C18A	1.523 (2)
N2—C7	1.3228 (16)	C17A—C20A	1.532 (3)
N2—C13	1.3881 (16)	C17A—H17A	0.9800
C1—C6	1.3908 (19)	C18A—C19A	1.520 (3)
C1—C2	1.3929 (19)	C18A—H18A	0.9700
C1—H1A	0.9300	C18A—H18B	0.9700
C2—C3	1.382 (2)	C19A—H19A	0.9600
C2—H2A	0.9300	C19A—H19B	0.9600
C3—C4	1.380 (2)	C19A—H19C	0.9600
C3—H3A	0.9300	C20A—H20A	0.9600
C4—C5	1.3857 (19)	C20A—H20B	0.9600
C4—H4A	0.9300	C20A—H20C	0.9600
C5—C6	1.4016 (18)	C17B—C20B	1.41 (3)
C5—H5A	0.9300	C17B—C18B	1.53 (3)
C6—C7	1.4743 (17)	C17B—H17B	0.9800
C8—C9	1.4002 (18)	C18B—C19B	1.45 (3)
C8—C13	1.4096 (17)	C18B—H18C	0.9700
C9—C10	1.3814 (18)	C18B—H18D	0.9700
C9—H9A	0.9300	C19B—H19D	0.9600
C10—C11	1.4076 (18)	C19B—H19E	0.9600
C10—H10A	0.9300	C19B—H19F	0.9600
C11—C12	1.3913 (17)	C20B—H20D	0.9600
C11—C14	1.4813 (18)	C20B—H20E	0.9600
C12—C13	1.3912 (17)	C20B—H20F	0.9600
C12—H12A	0.9300		
			
C14—O2—C15	115.59 (11)	O2—C14—C11	112.60 (11)
C8—N1—C7	106.08 (10)	O2—C15—C16	107.24 (12)
C8—N1—C17A	125.86 (11)	O2—C15—H15A	110.3
C7—N1—C17A	126.15 (11)	C16—C15—H15A	110.3
C8—N1—C17B	120.9 (7)	O2—C15—H15B	110.3
C7—N1—C17B	118.9 (7)	C16—C15—H15B	110.3
C17A—N1—C17B	21.9 (7)	H15A—C15—H15B	108.5
C7—N2—C13	104.52 (10)	C15—C16—H16A	109.5
C6—C1—C2	120.08 (13)	C15—C16—H16B	109.5
C6—C1—H1A	120.0	H16A—C16—H16B	109.5
C2—C1—H1A	120.0	C15—C16—H16C	109.5
C3—C2—C1	120.17 (14)	H16A—C16—H16C	109.5
C3—C2—H2A	119.9	H16B—C16—H16C	109.5
C1—C2—H2A	119.9	N1—C17A—C18A	109.82 (13)
C4—C3—C2	120.27 (13)	N1—C17A—C20A	112.10 (16)
C4—C3—H3A	119.9	C18A—C17A—C20A	113.48 (15)
C2—C3—H3A	119.9	N1—C17A—H17A	107.0
C3—C4—C5	120.06 (13)	C18A—C17A—H17A	107.0
C3—C4—H4A	120.0	C20A—C17A—H17A	107.0
C5—C4—H4A	120.0	C19A—C18A—C17A	112.42 (13)
C4—C5—C6	120.30 (13)	C19A—C18A—H18A	109.1
C4—C5—H5A	119.8	C17A—C18A—H18A	109.1
C6—C5—H5A	119.8	C19A—C18A—H18B	109.1
C1—C6—C5	119.11 (12)	C17A—C18A—H18B	109.1
C1—C6—C7	119.10 (11)	H18A—C18A—H18B	107.9
C5—C6—C7	121.73 (12)	C20B—C17B—C18B	113.7 (17)
N2—C7—N1	113.51 (11)	C20B—C17B—N1	121.4 (15)
N2—C7—C6	123.31 (11)	C18B—C17B—N1	100.4 (14)
N1—C7—C6	123.12 (11)	C20B—C17B—H17B	106.8
N1—C8—C9	132.38 (12)	C18B—C17B—H17B	106.8
N1—C8—C13	105.59 (11)	N1—C17B—H17B	106.8
C9—C8—C13	122.03 (12)	C19B—C18B—C17B	117 (2)
C10—C9—C8	116.52 (12)	C19B—C18B—H18C	108.1
C10—C9—H9A	121.7	C17B—C18B—H18C	108.1
C8—C9—H9A	121.7	C19B—C18B—H18D	108.1
C9—C10—C11	122.29 (12)	C17B—C18B—H18D	108.1
C9—C10—H10A	118.9	H18C—C18B—H18D	107.3
C11—C10—H10A	118.9	C18B—C19B—H19D	109.5
C12—C11—C10	120.68 (12)	C18B—C19B—H19E	109.5
C12—C11—C14	121.73 (11)	H19D—C19B—H19E	109.5
C10—C11—C14	117.59 (11)	C18B—C19B—H19F	109.5
C13—C12—C11	118.11 (11)	H19D—C19B—H19F	109.5
C13—C12—H12A	120.9	H19E—C19B—H19F	109.5
C11—C12—H12A	120.9	C17B—C20B—H20D	109.5
N2—C13—C12	129.33 (11)	C17B—C20B—H20E	109.5
N2—C13—C8	110.30 (11)	H20D—C20B—H20E	109.5
C12—C13—C8	120.36 (11)	C17B—C20B—H20F	109.5
O1—C14—O2	122.96 (12)	H20D—C20B—H20F	109.5
O1—C14—C11	124.44 (12)	H20E—C20B—H20F	109.5
			
C6—C1—C2—C3	−0.5 (2)	C14—C11—C12—C13	178.75 (12)
C1—C2—C3—C4	0.5 (2)	C7—N2—C13—C12	−178.75 (13)
C2—C3—C4—C5	0.2 (2)	C7—N2—C13—C8	−0.44 (14)
C3—C4—C5—C6	−0.8 (2)	C11—C12—C13—N2	179.43 (12)
C2—C1—C6—C5	−0.1 (2)	C11—C12—C13—C8	1.26 (18)
C2—C1—C6—C7	−177.39 (12)	N1—C8—C13—N2	0.35 (14)
C4—C5—C6—C1	0.76 (19)	C9—C8—C13—N2	−179.65 (12)
C4—C5—C6—C7	177.94 (12)	N1—C8—C13—C12	178.83 (12)
C13—N2—C7—N1	0.37 (15)	C9—C8—C13—C12	−1.2 (2)
C13—N2—C7—C6	177.83 (12)	C15—O2—C14—O1	2.0 (2)
C8—N1—C7—N2	−0.16 (15)	C15—O2—C14—C11	−178.34 (12)
C17A—N1—C7—N2	−165.11 (14)	C12—C11—C14—O1	−177.85 (13)
C17B—N1—C7—N2	−140.5 (8)	C10—C11—C14—O1	1.6 (2)
C8—N1—C7—C6	−177.63 (12)	C12—C11—C14—O2	2.47 (18)
C17A—N1—C7—C6	17.4 (2)	C10—C11—C14—O2	−178.11 (12)
C17B—N1—C7—C6	42.1 (8)	C14—O2—C15—C16	−170.51 (13)
C1—C6—C7—N2	43.20 (18)	C8—N1—C17A—C18A	−51.0 (2)
C5—C6—C7—N2	−133.98 (14)	C7—N1—C17A—C18A	111.06 (15)
C1—C6—C7—N1	−139.58 (13)	C17B—N1—C17A—C18A	33.3 (18)
C5—C6—C7—N1	43.24 (18)	C8—N1—C17A—C20A	76.11 (19)
C7—N1—C8—C9	179.88 (15)	C7—N1—C17A—C20A	−121.83 (15)
C17A—N1—C8—C9	−15.1 (2)	C17B—N1—C17A—C20A	160.4 (18)
C17B—N1—C8—C9	−40.8 (9)	N1—C17A—C18A—C19A	−49.37 (18)
C7—N1—C8—C13	−0.12 (14)	C20A—C17A—C18A—C19A	−175.71 (14)
C17A—N1—C8—C13	164.89 (14)	C8—N1—C17B—C20B	−44 (2)
C17B—N1—C8—C13	139.2 (9)	C7—N1—C17B—C20B	90.2 (18)
N1—C8—C9—C10	−179.62 (14)	C17A—N1—C17B—C20B	−154 (3)
C13—C8—C9—C10	0.4 (2)	C8—N1—C17B—C18B	82.2 (13)
C8—C9—C10—C11	0.3 (2)	C7—N1—C17B—C18B	−143.5 (10)
C9—C10—C11—C12	−0.1 (2)	C17A—N1—C17B—C18B	−27.8 (13)
C9—C10—C11—C14	−179.54 (13)	C20B—C17B—C18B—C19B	169 (2)
C10—C11—C12—C13	−0.65 (19)	N1—C17B—C18B—C19B	38 (2)

### Hydrogen-bond geometry (Å, °)

**Table:**

Cg1 is the centroid of the N1/N2/C7–C8/C13 ring.

**Table:**

*D*—H···*A*	*D*—H	H···*A*	*D*···*A*	*D*—H···*A*
C9—H9A···O1^i^	0.93	2.45	3.3781 (17)	172
C20A—H20B···O1^i^	0.96	2.56	3.419 (2)	148
C19A—H19B···Cg1	0.96	2.80	3.3793 (17)	120

Symmetry codes: (i) −*x*+1, *y*−1/2, −*z*+1/2.
